# Response to Buffoni

**DOI:** 10.1093/jncics/pkag105

**Published:** 2026-10-07

**Authors:** Jean S Edward

**Affiliations:** School of Nursing, University of Louisville, Louisville, KY, United States; Brown Cancer Center, University of Louisville, Louisville, KY, United States

We thank Dr Buffoni for his thoughtful commentary regarding our virtual oncology financial and legal navigation (OFLN) study. Dr Buffoni addresses important questions surrounding the global transferability of OFLN interventions and highlights how financial toxicity (FT) is inherently conditioned by the healthcare and socioeconomic environment in which cancer care is delivered.^1^

Although our trial was conducted within a multi-payer U.S. healthcare system, and more specifically in a rural context where direct and indirect medical costs^2^ and health insurance complexities are paramount, we agree that universal and publicly funded healthcare systems do not eliminate FT.^1^^,^^3^ In our study, frequently requested navigation services extended beyond direct financial assistance to encompass employment protections, school accommodations, and debt management. As Dr Buffoni notes, these indirect financial burdens also persist across single-payer and universal systems, highlighting the need for comprehensive support.^4^

Dr Buffoni also cautions that while OFLN digital delivery bypasses geographic barriers for rural populations, disparities in digital access and literacy could inadvertently deepen health inequities.^1^ Paradoxically, digital health interventions continue to expand as a primary means to enhance health literacy and, more specifically, health insurance literacy, particularly in oncology.^5^ In our study cohort, 61% of participants who resided in rural communities also experienced significantly greater improvements in post-intervention FT compared with their urban counterparts. To safeguard digital equity, we embedded proactive implementation strategies into our protocol as outlined in our publication: providing dedicated tablet devices (iPads) during clinic encounters, engaging clinic nurses to facilitate onboarding, and maintaining flexible, multimodal communications (phone, text, and email) alongside digital platforms. These proactive measures also enabled high enrollment (89%), retention (95%), and favorable participant evaluations across implementation outcomes of acceptability, appropriateness, and feasibility.^6^

Ultimately, we concur with Dr Buffoni that moving from proof-of-concept to broad scalability requires evaluating key implementation outcomes.^1^^,^^7^ To directly address these implementation and scalability concerns, our team is currently evaluating this OFLN model in a hybrid 1 implementation-effectiveness trial funded by a National Institutes of Health/National Cancer Institute R01 grant. This ongoing study will examine contextual implementation determinants and long-term effectiveness, offering essential insights to guide context-specific adaptation across diverse healthcare systems.

## Data Availability

No new individual-level data were generated or analyzed for this response.
